# Convergent Evolution of Unique Morphological Adaptations to a Subterranean Environment in Cave Millipedes (Diplopoda)

**DOI:** 10.1371/journal.pone.0170717

**Published:** 2017-02-08

**Authors:** Weixin Liu, Sergei Golovatch, Thomas Wesener, Mingyi Tian

**Affiliations:** 1 Department of Entomology, College of Agriculture, South China Agricultural University, Guangzhou, China; 2 Zoological Research Museum A. Koenig, Leibniz Institute for Terrestrial Biodiversity, Bonn, Germany; 3 Institute for Problems of Ecology and Evolution, Russian Academy of Sciences, Moscow, Russia; CNRS, FRANCE

## Abstract

Animal life in caves has fascinated researchers and the public alike because of the unusual and sometimes bizarre morphological adaptations observed in numerous troglobitic species. Despite their worldwide diversity, the adaptations of cave millipedes (Diplopoda) to a troglobitic lifestyle have rarely been examined. In this study, morphological characters were analyzed in species belonging to four different orders (Glomerida, Polydesmida, Chordeumatida, and Spirostreptida) and six different families (Glomeridae, Paradoxosomatidae, Polydesmidae, Haplodesmidae, Megalotylidae, and Cambalopsidae) that represent the taxonomic diversity of class Diplopoda. We focused on the recently discovered millipede fauna of caves in southern China. Thirty different characters were used to compare cave troglobites and epigean species within the same genera. A character matrix was created to analyze convergent evolution of cave adaptations. Males and females were analyzed independently to examine sex differences in cave adaptations. While 10 characters only occurred in a few phylogenetic groups, 20 characters were scored for in all families. Of these, four characters were discovered to have evolved convergently in all troglobitic millipedes. The characters that represented potential morphological cave adaptations in troglobitic species were: (1) a longer body; (2) a lighter body color; (3) elongation of the femora; and (4) elongation of the tarsi of walking legs. Surprisingly, female, but not male, antennae were more elongated in troglobites than in epigean species. Our study clearly shows that morphological adaptations have evolved convergently in different, unrelated millipede orders and families, most likely as a direct adaptation to cave life.

## Introduction

Caves represent one of the world’s most intriguing ecosystems [1,2], as, unlike surface habitats, they are completely devoid of sunlight, with neither photosynthesis nor plant growth, and have constant, usually much cooler temperatures and a limited food supply [3].

The cave environment is often separated into a twilight zone near the entrance, a middle zone of complete darkness with variable temperature, and a zone of complete darkness with nearly constant temperature in the deep interior [2]. The latter area is considered as the “true cave area” [4]. Each cave ecosystem is unique and often quite fragile; the peculiar cave-dwelling fauna are often characterized by their extreme scarcity and high endemism to a specific cave at the species level. The isolation and distinctiveness of each individual cave ecosystem, in combination with the locally evolved endemic cave species (troglobites: obligate cavernicoles), make caves important habitats for research in evolutionary adaptations [5,6].

The unique conditions inside caves and the unusual appearance of terrestrial arthropods adapted to a life inside them have captured the interest of the public and researchers alike [7–14]. The similarities in morphology of different cave arthropods have often been interpreted as examples of convergent evolution to similar ecological pressures [7,15–24]. However, recently this has been a subject of debate, as many of the presumed endemic cave taxa have been shown to have evolved not directly from surface-living (epigean) relatives, but from species already adapted to a special layer of the substrate, the so-called “Mesovoid Shallow Stratum” (MSS) [25,26] or “Shallow subterranean habitats” [27]. For example, some species that had previously been thought to be cave endemics were also discovered in the MSS [28–32].

Numerous aquatic and terrestrial arthropods are adapted to cave habitats [9,33–38]. Troglobitic species were previously thought to only evolve through climatic pressures, such as the Ice Age, or via the “cave refugium” hypothesis [39,40]. Therefore, endemic troglobitic species were considered to be absent from tropical countries. However, rich and diverse troglobites also occur in tropical and subtropical areas, especially in Asia [11,36,41–43].

Among the Myriapoda, carnivorous centipedes (Chilopoda) are rarely found in caves [44–46]. Detritivorous millipedes (Diplopoda) occur frequently in caves and form a diverse, sometimes dominant group of troglobites [3,42,47–52] that includes almost all major groups of millipedes [53], with species showing multiple, independent adaptions to a life in the cave ecosystem. Geographically, troglobitic millipede species are mainly known in Europe [54–57] and North America [51,52,58,59], but have also been recorded in South America [60], Africa [61], Australia [62] and, in the last 10–15 years, Asia [36,42,50,63,64].

**Fig 1:**
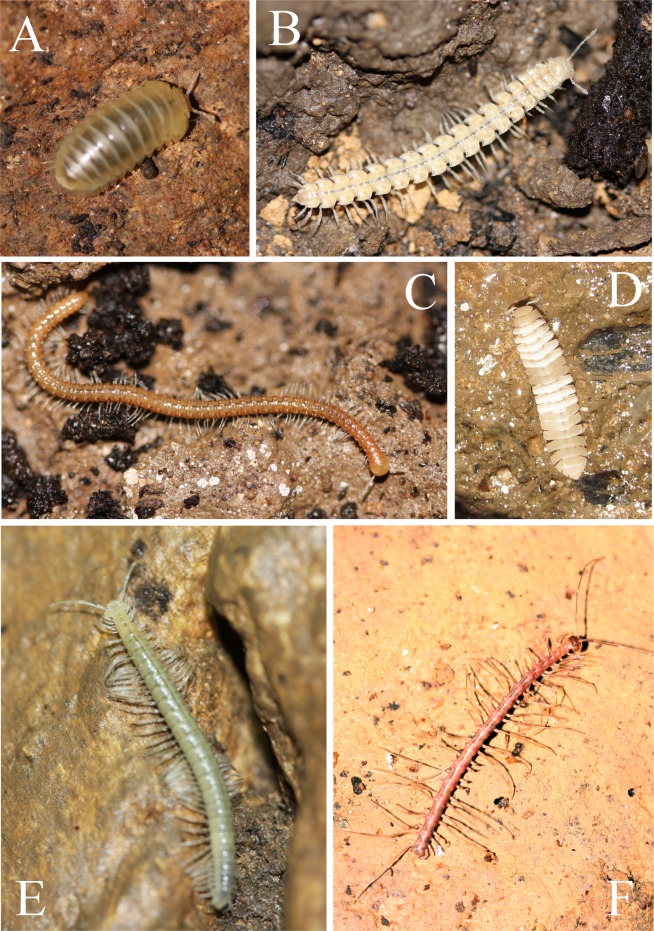
Photographs of troglobitic cave millipedes. **(A)** *Hyleoglomeris* sp. (Glomeridae, Glomerida); **(B)** *Epanerchodus* sp. (Polydesmidae, Polydesmida); **(C)** *Glyphiulus* sp. (Cambalopsidae, Spirostreptida); **(D)** *Eutrichodesmus* sp. (Haplodesmidae, Polydesmida); **(E)** *Nepalella* sp. (Megalotylidae, Chordeumatida); **(F)** *Desmoxytes* sp. (Paradoxosomatidae, Polydesmida).

The diversity, local endemism, and unusual appearance of cave millipedes (see Fig 1) have produced a rich taxonomic literature [50,65–68]. However, unlike taxonomic studies, there are only a few papers on the unusual and convergent morphological adaptations to cave life in Diplopoda [69–72]. For example, no study directly comparing a troglobite millipede with their epigean counterpart to find morphological adaptations to the cave environment has yet been conducted, most comparisons remain anecdotal [12].

During the last decade, the diversity of cave millipedes in China has been revealed. Currently, approximately 200 epigean millipede species from China are known. Between 2004 and 2016, about 100 millipede species were described from Chinese caves [63,64,66,67,73–91], with many more still awaiting description. However, not all of these species show the characters of true troglobites, and some may be shown to inhabit surface habitats. Troglobitic species in China belong to six orders and 13 different families. All troglobitic species belong to genera for which numerous epigean species are known, many of them recently described or redescribed from SE Asia [92–99]. These recent discoveries provide us with sufficient material to conduct the first comparative morphological study on the adaptations to cave life in millipedes. Species originating from southern Chinese karsts provide an additional opportunity, as the scarcity of MSS environments in this tropical to subtropical area of China means that morphological adaptations observed in cave millipedes are unique adaptations to a life in caves, rather than to a life in the MSS.

Here we compare different morphological characters in six troglobitic and epigean millipede species pairs, belonging to four different orders and six different families to identify general and convergently evolved morphological adaptations to the cave ecosystem.

## Material and Methods

### Species selection

**Fig 2:**
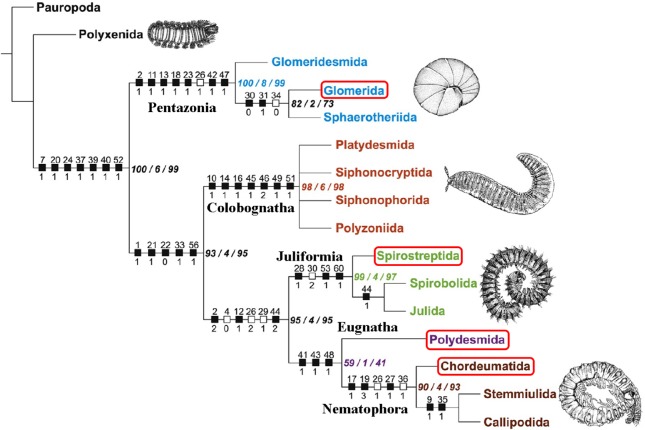
Phylogenetic tree and selected taxa. Species pairs included in this study are marked in red. Modified from [100].

Species of four orders (Glomerida, Polydesmida, Chordeumatida, and Spirostreptida), six families, and six genera (Fig 1) that represent the taxonomic diversity of Diplopoda (Fig 2) were selected based on availability of specimens and relatedness. Species from each genus were chosen randomly, based on the selection criteria that both conspecific male and female specimens were available.

All troglobitic species were collected from different limestone caves in south China (see Table 1). Some were paratype specimens of recently discovered species [90,91].

**Table 1:** Specimens selected and repositories of the vouchers. Troglobites are marked in bold. Abbreviations for museum repositories: MNHN = Muséum national d’histoire naturelle, Pairs, France; SCAU = South China Agricultural University, Guangzhou, China; SWUNM = Srinakharinwirot University Natural History Museum, Bangkok, Thailand; ZMUC = Zoological Museum, University of Copenhagen, Copenhagen, Denmark; ZMUM = Zoological Museum, Moscow State University, Moscow, Russia.

Order	Family	Species	Ecology (locality)	Repository/collection
Glomerida	Glomeridae	***Hyleoglomeris grandis* Liu and Tian, 2015**	Cave Qiaoqu, Guangxi, China	SCAU, 1 ♂, 1 ♀ paratypes
		*Hyleoglomeris* sp.	Thailand	SWUNM, 1 ♂, 1 ♀
Polydesmida	Paradoxosomatidae	***Desmoxytes phasmoides* Liu et al. 2016**	Cave Fengliu, Guangxi, China	SCAU, 1 ♂, 1 ♀ paratypes
		*Desmoxytes rubra* Golovatch and Enghoff, 1994	lowland rainforest, Yala, Thailand	ZMUC, 1 ♂, 1 ♀ paratypes
Polydesmida	Polydesmidae	***Epanerchodus* sp**.	Cave Zhakou, Hubei, China	SCAU, 1 ♂, 1 ♀
		*Epanerchodus* sp1.	Yunnan, China	ZMUM, 1 ♂
		*Epanerchodus* sp2.	Yunnan, China	ZMUM, 1 ♀
Polydesmida	Haplodesmidae	***Eutrichodesmus planatus* Liu and Tian, 2013**	Cave Zhenzhuyan, Guangxi, China	SCAU, 1 ♂, 1 ♀
		*Eutrichodesmus* sp.	Sichuan, China	SCAU, 1 ♂, 1 ♀
Chordeumatida	Megalotylidae	***Nepalella* sp1.**	Cave Hejia, Guizhou, China	SCAU, 1 ♂, 1 ♀
		*Nepalella* sp2.	Sichuan, China	ZMUM, 1 ♂, 1 ♀
Spirostreptida	Cambalopsidae	***Glyphiulus* sp1.**	Cave Shuilian, Hubei, China	SCAU, 1 ♂, 1 ♀
		*Glyphiulus* sp2.	Jiangxi, China	SCAU, 1 ♂, 1 ♀

All epigean species used in this study were deposited vouchers in internationally accessible museum collections (Table 1), and chosen according to availability. Ideally, epigean sister taxa to the troglobitic species would have been included; however, no phylogeny for our investigated genera is available, although we note that the morphological characters utilized here show very little interspecific variation among congeneric epigean species, and most were not even mentioned in taxonomic descriptions [66,79,91,92,98].

### Character selection

**Fig 3:**
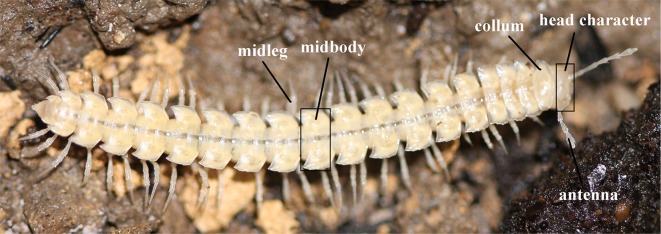
*Epanerchodus* sp. (Polydesmidae, Polydesmida). Morphological characters selected to compare cave and epigean millipede species.

Morphological characters were chosen based on the literature, and from our own observations. A detailed discussion of each character is provided in Table 2. We sampled characters from different body regions, such as the head or legs of millipedes (Fig 3). Characters were selected a priori. Unfortunately, in many cases, characters could not be scored in all six Diplopoda families as some, such as the organ of Tömösváry, were not present in all groups [101]. A total of 30 characters was selected for the analysis (Table 2), of which 10 could only be scored in a single or few families. Twenty characters were analyzed in all families.

**Table 2:** Character discussion.

**Fig 4:**
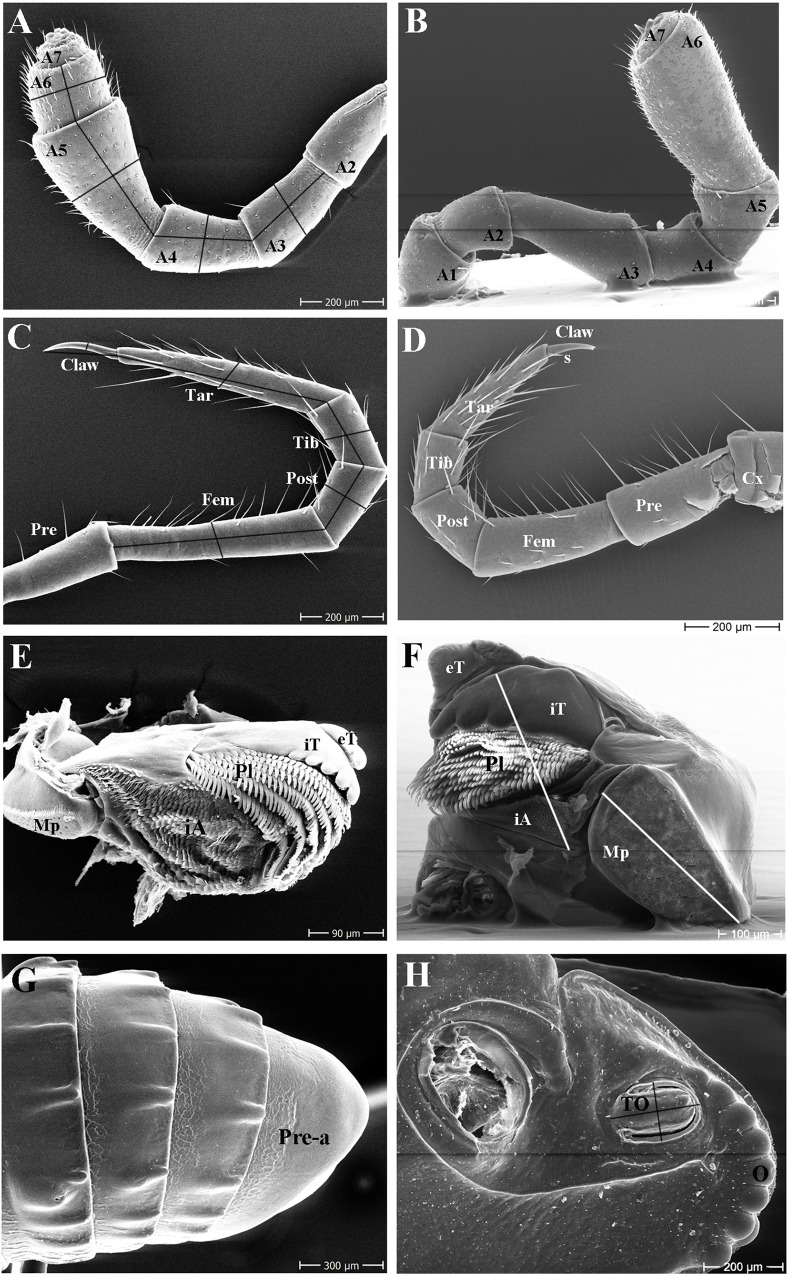
SEM plate measurements. **(A)** Antenna of a troglobitic *Glyphiulus* sp., antenna measurements, C8–C12; **(B)** Antenna of an epigean *Hyleoglomeris* sp., C13; **(C)** Midleg of a troglobitic *Glyphiulus* sp., midleg measurements, C24–C28; **(D)** Midleg of an epigean *Glyphiulus* sp., C29; **(E)** Mandible of a troglobitic *Glyphiulus* sp., C16–C17; **(F)** Mandible of an epigean *Hyleoglomeris* sp., mandible measurements, C18; **(G)** Telson of a troglobitic *Glyphiulus* sp., C30; **(H)** Head of an epigean *Hyleoglomeris* sp., number and size of ocelli, C4, C6; Tömösváry organ’s measurements, C7. **Abbreviations:** A1–A7 = antennomeres 1–7; Cx = coxa; Pre = prefemur; Fem = femur; Post = postfemur; Tib = tibia; Tar = tarsus; s = accessory spine; eT = external tooth; iT = internal tooth; Pl = pectinate lamellae; iA = intermediate area; Mp = molar plate; Pre-a = Pre-anal; TO = Tömösváry organ; O = ocelli.

Characters
**C1.** *Body with color/pigmentation*: according to the literature, troglobites often lose their pigmentation [12] (Fig 1).
**C2.** *Body length (mm)*: some studies show that some troglobitic species have shorter bodies [70]. Body length was measured from head to telson.
**C3.** *Midbody width (mm)*: midbody width was measured from left middle–lateral to right middle–lateral margin (Fig 3).
**C4.** *No*. *of ocelli*: numerous taxonomic studies show a reduced number of ocelli in cave millipede taxa [82–84,90]. Generally, Glomerida possess a lower number of ocelli (Fig 4H); while epigean species in the Chordeumatida and Spirostreptida usually have a relatively high number of ocelli [102]. Polydesmida generally have no eyes [101], therefore, this character could not be scored in the three families of the Polydesmida.
**C5.** *Color of ocelli*: in taxonomic descriptions of troglobitic millipedes, the color of ocelli is often described as much lighter in troglobites than in epigean species [82,84,90]. This character could not be scored in the three families of the Polydesmida, as they have no eyes.
**C6.** *Size of ocelli*: in taxonomic descriptions of millipedes, the ocelli are often described as reduced in size [84,90] and nearly obliterated in troglobitic species [82,83]. This character could not be scored in the three families of the Polydesmida, as they have no eyes.
**C7.** *Tömösváry organ*, *length/width ratio*: the Tömösváry organ is present and horseshoe-shaped [103] (Fig 4H) only in the Glomerida, whereas this character was described to differ between cave taxa and epigean congeners [69]. This character was not measured in the Polydesmida, Chordeumatida, or Spirostreptida, as they lack such an organ [100].
**C8.** *Antenna*, *antennomere 3*, *length/width ratio*: in taxonomic descriptions of cave millipedes, it is often assumed that cave species have a more elongated antenna than epigean species [76,88,91]. Therefore, the length/width ratio of antennomere 3 was measured as illustrated (Fig 4A).
**C9.** *Antenna*, *antennomere 4*, *length/width ratio*: see above; the length/width ratio of antennomere 4 was measured as illustrated (Fig 4A).
**C10.** *Antenna*, *antennomere 5*, *length/width ratio*: see above; the length/width ratio of antennomere 5 was measured as illustrated (Fig 4A).
**C11.** *Antenna*, *antennomere 6*, *length/width ratio*: see above; the length/width ratio of antennomere 6 was measured as illustrated (Fig 4A).
**C12.** *Antenna*, *antennomere 3*–*6*, *length/width ratio*: similar to characters 8–11, the sum of length/sum of width ratio of antennomeres 3–6 was also calculated.
**C13.** *Antenna*, *antennomere 6 maximal width*: the maximal width of antennomere 6, which bears the apical disc (also referred to as antennomere 7), is usually located near the apical tip (Fig 4A); according to the literature, this character differs in some troglobites [72] (Fig 4B).
**C14.** *Antenna*, *antenna apical cones*: the apical disc often carries four visible, long apical cones (Fig 4B), but in our study, these apical cones were modified in some cave species (Fig 4A).
**C15.** *Labrum tooth*: Glomerida species carry a single tooth; Polydesmida and Chordeumatida carry three central teeth; and Spirostreptida have 3–6 teeth [67,82]. According to the literature, the number of labral teeth is sometimes reduced in troglobites [70].
**C16.** *Mandible*, *external tooth*: the millipede mandible generally carries a single external tooth [100] (Fig 4E and 4F); however, we discovered some millipede species with a different number. As cave millipedes have a different food source than epigean species, we added this and the following mandible characters to see if there was a general difference in the mandible between epigean and troglobitic millipedes.
**C17.** *Mandible*, *number of cusps of internal tooth*: the internal tooth consists of several cusps (Fig 4E and 4F); we discovered some modification of this character in the Spirostreptida species.
**C18.** *Mandible*, *correlation of size of pectinate lamellae plus intermediate area with the size of the molar plate*: according to the literature, the mandible pectinate lamellae (Fig 4E and 4F) are hypertrophied in some troglobitic species [70]. Here we measured the length of pectinate lamellae, plus intermediate area, divided by the size of the molar plate to obtain the ratio (Fig 4F).
**C19.** *Collum*, *length/width ratio*: the collum is used for digging in some epigean millipede species, a function that may no longer be necessary in cave millipedes. The length/width ratio of the collum was measured.
**C20.** *Development of collum crests*: taxa of the order Spirostreptida feature unusual crests on the collum [66,82]. We included this character as the collum might have a different function in cave taxa. Crests were only present in the Spirostreptida, therefore, this character not be scored in any other family.
**C21.** *Shape of the paraterga*: the paraterga are known to be modified only in *Desmoxytes* (Polydesmida, family Paradoxosomatidae). Troglobitic cave species of the Polydesmida family Paradoxosomatidae were described as having long-spiniform paraterga, while epigean species have more wing-like ones [91]. This character was only scored in the family Paradoxosomatidae, order Polydesmida.
**C22.** *3+3 setae on metaterga*, *length*: species of the order Chordeumatida have a characteristic arrangement of three setae (Fig 1E) on each side of the tergites [100]. As these setae have a potential sensory function, we investigated whether the length of these setae varied between epigean and troglobitic species. This character was not applicable to the other millipede orders.
**C23.** *Development of metazonae crests*: the crests on the metazonae [66,82] (Fig 1C) are only present in the Spirostreptida, therefore, this character was not scored in any other family.
**C24.** *Midleg*, *femur*, *length/width ratio*: taxonomic descriptions often mention that troglobitic millipede species have elongated legs than those of their epigean counterparts [76,78,80]. The length/width ratio of the femur was measured as illustrated (Fig 4C).
**C25.** *Midleg*, *postfemur*, *length/width ratio*: see above; the length/width ratio of the postfemur was measured as illustrated (Fig 4C).
**C26.** *Midleg*, *tibia*, *length/width ratio*: see above; the length/width ratio of the tibia was measured as illustrated (Fig 4C).
**C27.** *Midleg*, *tarsus*, *length/width ratio*: see above; the length/width ratio of the tarsus was measured as illustrated (Fig 4C).
**C28.** *Midleg*, *claw*, *length/width ratio*: see above; the length/width ratio of the claw was measured as illustrated (Fig 4C).
**C29.** *Midleg*, *claw length/accessory spine length ratio*: a midleg tarsus claw with an accessory spine was only present in the Spirostreptida [66,82] (Fig 4C and 4D). The claw length/accessory spine length ratio was measured. This character could not be applied to any other family.
**C30.** *Pre-anal crest*: the pre-anal ring often carries a crest in the Spirostreptida [66,82], but in our study they appeared to be modified in cave species (Fig 4G). This character was not applicable in other families.

### Character measurements

In order to avoid a size bias, only the general size of the specimen was measured in millimeters. All other measurements were objective and are illustrated (Fig 4). Scanning electron microscopy images, as well as camera lucida line drawings were used to measure the length/width ratio of the different characters.

### Character analyses

A description of all characters is provided in Table 2. Male Diplopoda often possess longer legs and antennae than conspecific females. To avoid this sexual bias in our study, male and female characters were scored separately, i.e., a male troglobite was only compared with a male epigean specimen and vice versa. One exception was the family Polydesmidae, genus *Epanerchodus*, for which we could not obtain a female epigean specimen. Altogether, 60 character pairs (30 for each genus/sex) were compared.

All characters were measured and recorded using Microsoft Office Excel (version 2010) (see S1 and S2 Tables). Measured character/state pairs were directly visually compared. Of the 30 characters, 10 could not be measured in all six families, but were nevertheless included to provide the basis for future studies with more millipede groups.

## Results

### Convergently evolved cave adaptations in millipedes

**Fig 5:**
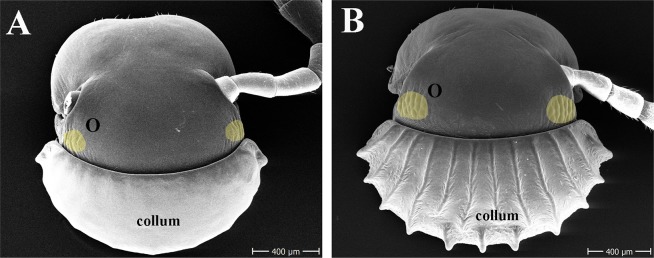
SEM plate of *Glyphiulus* spp. **(A)** Head and collum of a troglobitic *Glyphiulus* sp.; **(B)** Head and collum of an epigean *Glyphiulus* sp. **Abbreviations:** O = ocelli.

Ten characters could not be scored in all six families. Seven were only present in a single family. The Tömösváry organ (c7) was only present in the Glomerida; the shape of the paraterga (c21) was only modified in the family Paradoxosomatidae (Polydesmida); 3+3 setae on the metaterga (c22) were only present in the order Chordeumatida. Four characters, the crest(s) on the collum (Fig 5), the metazonae, the pre-anal ring (c20, 23, 30), and a modified apical spine on the walking legs (c29), were only present in the family Cambalopsidae (Spirostreptida). Three characters related to the eyes were absent from the three families in the order Polydesmida. The number (c4) and color of ocelli (c5) were reduced in all troglobitic millipede species that had eyes (Fig 5). The size of the ocelli (c6) was smaller in the Glomerida and the family Cambalopsidae, but no difference was observed in this character between epigean and troglobitic species in the Chordeumatida.

Twenty characters were analyzed in all six Diplopoda families. Two characters, the width of the midbody segments (c3) and the collum (c19), did not differ in most of the compared troglobitic/epigean species pairs (see S1 and S2 Tables). Troglobitic species had a slenderer midbody body ring width than their epigean congeners in the family Polydesmidae (Polydesmida). In contrast, troglobitic species were wider than their epigean counterparts in the families Paradoxosomatidae and Haplodesmidae (Polydesmida).

Five of the 20 characters only differed in one of the six families between epigean and troglobitic species pairs. The maximal width of antennomere 6 (c13) only differed in the Glomerida; only the Spirostreptida showed several cephalic characters (c15–17) and differently shaped antennal apical cones (c14).

Nine of the 20 characters were ambiguous, as they were distinct between some troglobitic and epigean species, but not in all families. For example, antennomeres 3–6 (c8–12) were more elongated in troglobitic species from four of the families, but not in the Polydesmidae, or Spirostreptida. The molar plate of the mandible was smaller in troglobitic species than the part covered by the pectinate lamellae (c18), except in the Polydesmidae. The postfemur, tibia, and claw of walking legs (c25, 26, 28) was elongated in many troglobitic species, but not in the species from the families Polydesmidae or Paradoxosomatidae, nor in the Glomerida.

**Fig 6:**
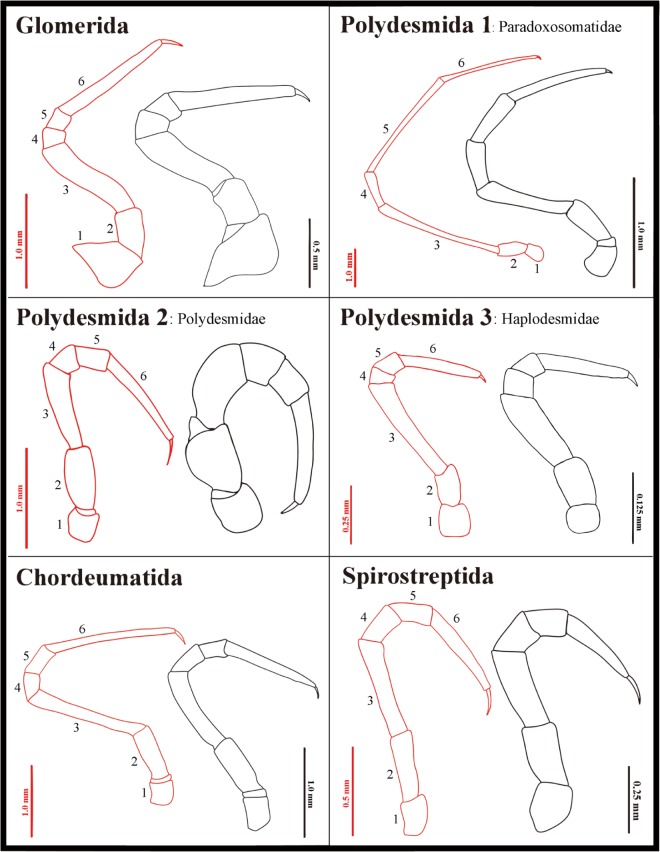
Male midbody legs of each of the six families. Red color represents the leg of a troglobite; black color marks the epigean congener. 1: coxa; 2: prefemur; 3: femur; 4: postfemur; 5: tibia; 6: tarsus.

Four characters differed in all troglobitic/epigean species pairs. Two of the characters were body color (c1) and body length (c2). Troglobites were much lighter and slightly larger than their epigean congeners. For the other two characters, the femora (c24) and tarsi (c27), but not other leg joints, were more strongly elongated in troglobitic millipedes than in their epigean counterparts (Fig 6).

### Sexual differences

**Fig 7:**
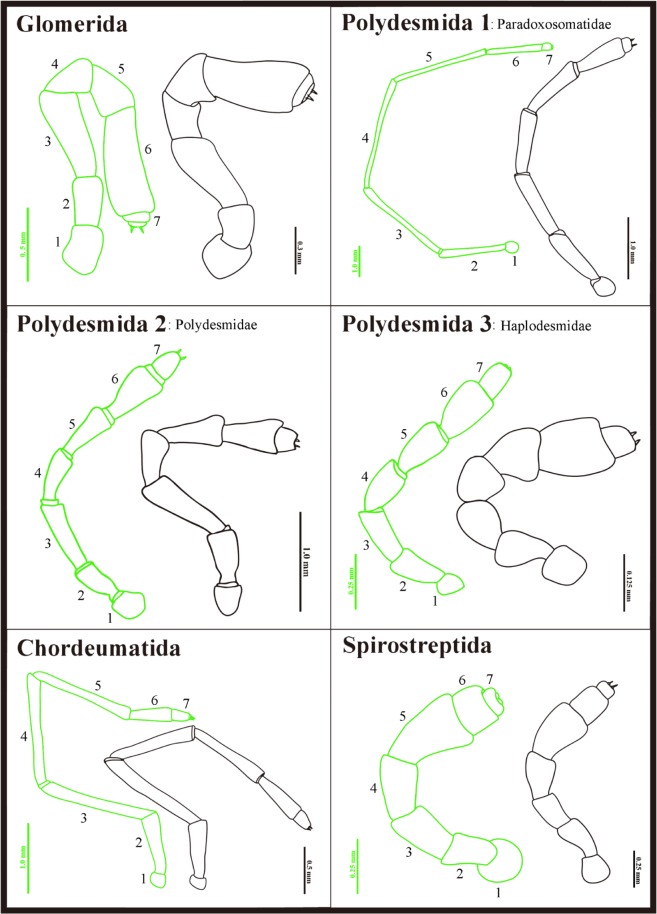
Male antennae in each of the six families. Green color represents the troglobite species; black color the epigean species. 1–7: antennomeres 1–7. Differences in the length/width ratio of the antennomeres were only obvious in the family Paradoxosomatidae (Polydesmida).

Generally, there were little sex based differences in morphological characters as the differences observed in the 30 studied morphological characters between troglobitic and epigean millipede species were identical in males and females (see S1 and S2 Tables). However, antennomere 3 (c8) was always more elongated in female troglobitic species; the length/width ratio of antennomeres 3–6 (c12) was greater in troglobitic female millipedes than in epigean females, but neither of these differences were observed in their male conspecifics (Fig 7).

## Discussion

### Morphological character adaptations to cave life present in single Diplopoda orders

Only 12 characters differed between troglobitic and epigean species within a single order. In the pill millipedes of the order Glomerida, the Tömösváry organ (Table 2: c7) in troglobites was slenderer and longer than in epigean species. Tabacaru [69] also found that the slit of the Tömösváry organ was wider in troglobitic pill millipedes of the genus *Trachysphaera* than in epigean congeners from Romania and was also differently ornamented. Golovatch and Enghoff [72] mentioned that the maximal width of antennomere 6 and the length/width ratio of the Tömösváry organ might represent potential synapomorphy of the *Glomeris alluaudi* group, which contains several presumed troglobites, on the Canary Islands. While this character has not been mentioned in the taxonomic descriptions of many cave pill millipede species, the maximal width of antennomere 6 (Table 2: c13) was found at the apex in our troglobitic *Hyleoglomeris* species, but centrally in its epigean counterpart. Both the organ of Tömösváry and the antennae are clearly of a sensory function.

Our species pair in the family Paradoxosomatidae, order Polydesmida, showed the same adaptations as those recorded in the literature [91]. Our troglobitic species of the genus *Desmoxytes* possessed modified paraterga (long and spiniform) (Fig 1F), while the epigean species of the same genus had wing-shaped paraterga (Table 2: c21). The function of the paraterga in Polydesmida species has not been analyzed; they could provide protection at the base of the legs against predators, or enhance the spread of the poisonous defense fluid released by species in the order [104]. Both are defensive functions that might be modified or lost in species adapted to the cave environment.

In the Chordeumatida, the 3+3 setae on the metaterga (Table 2: c22) were much shorter in the troglobitic species of the genus *Nepalella* than in their epigean counterparts. We have no explanation for this observation, as the setae are thought to have a sensory function that might be useful in a cave environment. As the Chordeumatida are a common and species-rich order of cave fauna in North America and Europe [47,52,57,65], this character should be studied in other families of the order to see if a shortening of these characteristic setae is a common adaptation to a troglobitic life.

In the species of the Cambalopsidae, order Spirostreptida, numerous differences were observed between epigean and cave species of the genus *Glyphiulus*; none of the characters were observed in any other millipede family. The antennal apical cones (Table 2: c14) were much shorter in troglobitic species of *Glyphiulus*. The number of labral teeth (Table 2: c15), as well as the number of external (Table 2: c16) and internal teeth (Table 2: c17) on the mandible were reduced in troglobitic *Glyphiulus* species. These mandible characters could be related to the different food sources available to troglobitic *Glyphiulus* species that are known consumers of bat guano [42], which is a much softer food than leaf litter. Troglobitic *Glyphiulus* also differed from epigean congeners by the presence of flat, nearly obliterated crests on the collum (Table 2: c20), the metazonae (Table 2: c23) and the pre-anal ring (Table 2: c30). These crests probably fulfill a defense function that might no longer be necessary in the cave environment, where vertebrate predators are rare or entirely absent. In addition, the claw in troglobitic *Glyphiulus* species was larger than its accessory spine (Table 2: c29).

Based on these highlighted potential order- or family-specific cave adaptations, future studies focusing on a single taxonomic group might discover if these characters are indeed common cave adaptations in these groups or artifacts of our randomized species sampling.

### Convergent cave adaptations observed in Chinese Diplopoda compared to those of earlier studies

Enghoff [70,71] studied the mouthparts of semi-aquatic cave millipedes, which evolved convergently in troglobitic European species belonging to the orders Polydesmida and Julida. The mandibles are modified to filter food from cave streams, similar to Baleen whales [105]. Such adaptations were not observed in any of our studied millipede species from Asia.

Our study is the first to compare a diverse set of characters in different troglobitic millipede species with those in congeneric epigean species reflecting the diversity of the Diplopoda (four orders and six families) and including representatives of both sexes.

### Elongation of the antennae as an adaptation to a troglobitic life

The relative antennal length and the number of antennomeres are generally treated as troglomorphic characters that are more elongated with an increased adaptation to subterranean life [106]. Therefore, our discovery that the antennae were not more elongated in the troglobites than in epigean millipede species was surprising. However, Culver et al. [20] observed that epigean populations of the aquatic amphipod *Gammarus minus* Say, 1818 possess longer antennae than populations of the same species living in troglobitic habitats. In other amphipod species of the genus *Stygobromus* Cope, 1872, no difference in antennal length was found between epigean and troglobitic species [107]. In pill millipedes of the genus *Trachysphaera*, clearly more elongated antennae were described in cave species than in epigean species [69]. In our study, the male antennae were elongated only in four of the six millipede families analyzed: weekly in Glomeridae, Megalotylidae, and Haplodesmidae, and strongly elongated in the Paradoxosomatidae (Fig 7). They were not elongated either in the Cambalopsidae or in the Polydesmidae. Therefore, antennal elongation appears to be a cave adaptation present in many cave millipede species, but not a general adaptation to cave life. Antennae are an important sense organ to gather environmental information [108], such as finding mating partners, food sources, or predators. We have no clear explanation for the fact that only the female antennae were more elongated than those of their epigean counterparts in our study. A comparative examination of the antennal length in different individuals of the troglobitic millipede taxa should be undertaken in the future, although we caution that specimens of cave millipede species are scarce, with only very limited numbers of specimens being available in collections.

### Cave adaptations or MSS adaptations?

The "climatic relict hypothesis" [3] suggests that many troglobites have evolved from surface-dwelling ancestors that sought refuge from climatic stress, notably, during the periods of Quaternary glaciations. However, other factors may have been involved in the colonization of caves by millipedes, especially in tropical areas. For example, many troglophilic and troglobitic Spirostreptida of the family Cambalopsidae from southeastern Asia are strongly associated with the presence of bat guano [42], and this food source may have been as important as the buffered climatic conditions in caves in determining their colonization success.

Our results show that the body and ocelli of troglobites were much lighter than in epigean species, with a strongly reduced number of ocelli in troglobites. However, these character traits can also be observed in the few known Diplopoda species that inhabit the MSS, such as *Ostinobolus subterraneus* Wesener, 2009 (Spirobolida) or *Propolydesmus germanicus* Verhoeff, 1896 (Polydesmida). Unfortunately, no clear MSS species are currently known from the genera studied here, as this habitat type is rare in South China; therefore, no MSS species were included in our analysis. However, the observed elongation of the femora and tarsi in troglobitic millipede species appears to be a unique morphological adaptation to a life in the cave ecosystem. Generally, MSS millipede species have shorter legs than their epigean counterparts [30,109]. The elongated legs of cave millipedes could be directly related to their underground lifestyle. Most troglobitic millipede species are observed walking on the walls of the cave, while epigean millipede species are often found buried in leaf litter, while MSS species “swim” within the soil. This “open” style of living of troglobitic species might reduce the evolutionary pressure towards shorter legs, or make locomotion with longer legs energetically more beneficial [110,111]. Why only the femora and tarsi, but not the other podomeres, were elongated in troglobitic species in our study remains unknown.

Cave gigantism is not necessarily viewed as a troglomorphic feature and this character was not included in recent lists of general troglobitic characters of other cave fauna [17,112]. Our study indicates that troglobitic millipedes were larger than their epigean counterparts, which might be related to the fact that there are no space constraints and few predators in the cave ecosystem [113]. In our study, the measurement of body length in the pill millipede species (Glomerida) was difficult as the specimens were partly rolled up. Comparisons of more troglobitic millipede species with their epigean counterparts should be conducted in the future to examine whether “cave gigantism” is restricted to our particular species pairs, or represents a general characteristic/trend.

### Problems with the comparison of characters

Mandibular characters are rarely explored in millipedes [100,114], but already show promising variations in some studied cave millipede species [70,71]. However, in our study the length and width ratio of different parts of the mandible proved to be especially difficult to compare because of the different angles of observation and these results should, therefore, be treated with caution [115]. However, the number of labral teeth was generally quite consistent in the Diplopoda [116]. Only the troglobitic species of Cambalopsidae (order Spirostreptida) had a reduced number of labral teeth (see above).

### Cave adaptations in millipedes compared to those of other terrestrial arthropod taxa

For terrestrial arthropods, similar environmental pressures are expected to produce similar external morphological adaptation in taxa to living a troglobitic life. For example, Opiliones (Chelicerata) show numerous convergent adaptations to the cave ecosystem [117,118], including a reduction or absence of eyes, increased length of legs, and reduced pigmentation/sclerotization. Cave adaptations and potentially troglomorphic convergent characters were also studied in the Arachnida, especially species of the genus *Anthrobia* Tellkampf, 1844 [22]. Within this genus, troglobites exhibited the following putative adaptations to cave life: loss of eyes, elongation of the legs, and reduction of the tracheal system. The tergal cuticle of the troglobitic terrestrial isopod *Titanethes albus* Koch, 1841 was analyzed by Hild et al. [23]. They found that cave species had a poorer resistance against water loss from the epicuticle and a low mechanical strength and rigidity of the cuticle as compared to epigean species.

Among the insects, Coleoptera also have numerous species with morphological and anatomical adaptations to cave life [119]. The evident morphological changes in cave beetles include the reduction or complete lack of eyes, the loss of pigmentation, a thinner cuticle, fused elytra, as well as elongation of the body, antennae, and legs, which become longer and slenderer. The internal anatomical modifications include huge vesicles in the fat body, and absent or smaller unicellular glands. Therefore, in other troglobitic terrestrial arthropod groups, the adaptations also include many internal modifications. In order to get better insights in the adaptations to cave life in animals, all these character complexes should be studied in millipedes in the future.

### Future directions

Our study focused mainly on external morphological characters. As mentioned above, our dataset should be expanded to include internal morphological characters, such as the tracheal system [22] and the thickness of the cuticle [23]. The scarcity of cave specimens currently prevents such invasive studies, but with the advent of non-invasive CT-technology [100,120] such research might be possible in the future.

In addition, our study focused on SE Asian cave millipedes and their epigean counterparts. The much more diverse (or better known) North American and European cave millipede faunas should also be studied to examine whether the general morphological adaptations observed in SE Asian genera and families also occur in other taxa of the Diplopoda. Ideally, phylogenetic analyses would be conducted using better-accessed European millipede genera, which would allow a direct comparison of potential epigean/troglobitic sister taxa.

During the last decade, molecular data have become available for subterranean taxa and their corresponding surface relatives, which has improved insights into the evolution of cave fauna [121]. Finally, some transcriptomes are available for cave-dwelling animals, including a cave beetle species, an aquatic isopod crustacean, and three different species of cave fish [122]. Comparing transcriptomes of troglobitic millipedes with those of epigean congeners might provide additional evidence of the genetic pathways that contribute to their survival and evolution in the unusual ecosystem of the cave.

## Supporting Information

S1 Table Male characters. (DOCX)

S2 Table Female characters. (DOCX)

## References

[1] Barr TC. Observations on the ecology of caves. Am Nat 1967; 101 (922): 475–491.

[2] Poulson TL, White WB. The cave environment. Science 1969; 16 (3897): 971–981. 10.1126/science.165.3897.971 17791021

[3] Culver DC, Pipan T. The biology of caves and other subterranean habitats. Oxford, New York. 2009.

[4] Tuttle MD, Stevenson DE. An analysis of migration as a mortality factor in the gray bat based on public recoveries of banded bats. Am Midl Nat 1977; 235–240.

[5] Juberthie C. Underground habitats and their protection. Council of Europe 1995.

[6] Whitten T. Applying ecology for cave management in China and neighbouring countries. J Appl Ecol 2008; 46 (3): 520–523.

[7] Barr TC. Cave ecology and the evolution of troglobites Evolutionary Biology. New York: Plenum; 1968 p. 33–102.

[8] Lamprecht G, Weber F. The regressive evolution of the circadian system controlling locomotion in cavernicolous animals. On the evolution of behavior in carabid beetles. Veenman and Zonen, Wageningen 1979; 69–82.

[9] Barr TC, Holsinger JR. Speciation in cave faunas. Annu Rev Ecol Syst 1985; 16 (1): 313–337.

[10] Sket B. Why all cave animals do not look alike–a discussion on adaptive value of reduction processes. The Nss Bull 1985; 47 (2): 78–85.

[11] Deharveng L, Bedos A. The cave fauna of Southeast Asia. Origin, evolution and ecology. Ecosystems of the world 2000; 603–632.

[12] Harvey MS, Shear WA, Hoch H. Onychophora, Arachnida, Myriapods and Insecta. In: Wilkens H, Culver DC, Humphreys WF, editors. Subterranean Ecosystems. Ecosystems of the world 30; 2000. p. 79–94.

[13] Culver DC, Deharveng L, Bedos A, Lewis JJ, Madden M, Reddell JR, et al The mid-latitude biodiversity ridge in terrestrial cave fauna. Ecography 2006; 29 (1): 120–128.

[14] Pricop E, Negrea BM. On the adaptations to cave life of some different animal groups (first note). Extreme Life Biospeology & Astrobiol 2009; 1 (2): 41–47.

[15] Barr TC. The cavernicolous beetles of the subgenus *Rhadine*, genus *Angonum* (Coleoptera: Carabidae). Am Midl Nat 1960; 45–65.

[16] Chirstiansen K. Convergence and parallelism in cave Entomobryinae. Evolution 1961; 15 (3): 288–301.

[17] Chirstiansen K. Morphological adaptations In: White WB, Culver DC, editors. Encyclopedia of caves, 2nd ed. Academic/Elsevier; 2012 p. 517–528.

[18] Howarth FG. Ecology of cave arthropods. Annu Rev Entomol 1983; 28: 365–389.

[19] Culver DC, Fong DW. Why all cave animals look alike. Stygologia 1986; 2: 208–216.

[20] Culver DC, Kane TC, Fong DW. Adaptation and natural selection in caves: the evolution of *Gammarus Minus*. Harvard University; 1995.

[21] Moldovan OT, Jalžić B, Erichsen E. Adaptation of the Mouthparts in some subterranean Cholevinae (Coleoptera, Leiodidae). Nat Croat 2004; 13 (1): 1–18.

[22] Miller JA. Cave adaptation in the spider genus *Anthrobia* (Araneae, Linyphiidae, Erigoninae). Zool Scr 2005; 34: 565–592.

[23] Hild S, Neues F, Žnidaršič N, Štrus J, Epple M, Marti O, et al Ultrastructure and mineral distribution in the tergal cutile of the terrestrial isopod *Titanethes albus*. Adaptation to a karst cave biotope. J Struct Biol 2009; 168: 426–436. 10.1016/j.jsb.2009.07.017 19632333

[24] Pipan T, Culver DC. Convergence and divergence in the subterranean realm: a reassessment. Biol J Linn Soc 2012; 107 (1):1–14.

[25] Juberthie C, Delay B. Bouillon M. Extension du milieu souterrain en zone non calcaire: description d'un nouveau milieu et de son peuplement par les Coléoptères troglobies. Mémoires de biospéologie 1980; 7: 719–752.

[26] Racovită E. Essay on biospeological problems (Culver DC and Moldavan OT [trans.]) Emil George Racovitza. Essay on biospeological problems. Bucharest: Casa Carti de Stiintap 2006; 127–183.

[27] Culver DC, Pipan T. Shallow subterranean habitats: ecology, evolution, and conservation. Oxford University; 2014.

[28] Giachino PM, Vailati D. The subterranean environment: hypogean life, concepts and collecting techniques. WBA Handbooks 2010; 3:1–132.

[29] Schönhofer AL, Martens J. The enigmatic Alpine opilionid *Saccarella schilleri* gen. n., sp. n. (Arachnida: Nemastomatidae)–isolated systematic placement inferred from comparative genital morphology. Org Divers Evol 2012; 12 (4): 409–419.

[30] Enghoff H. Three new species of *Dolichoiulus* millipedes from the underground of Gran Canaria, with notes on the circumscription of the genus (Diplopoda, Julida, Julidae). Eur J Taxon 2012; 15:1–12.

[31] Enghoff H. New montane, subterranean congeners of a littoral millipede, genus *Thalassisobates* (Diplopoda: Julida: Nemasomatidae). J Nat Hist 2013; 47 (23–24): 1613–1625.

[32] Enghoff H, Reboleira ASPS. Subterranean species of *Acipes* Attems, 1937 (Diplopoda, Julida, Blaniulidae). Zootaxa 2013a; 3652 (4): 485–491. 26269848 10.11646/zootaxa.3652.4.6

[33] Poulson TL. Cave adaptation in amblyopsid fishes. Am Midl Nat 1963; 257–290.

[34] Reddell JR. The cave fauna of Texas with special reference to the western Edwards Plateau In: Elliott WR, Veni G, editors. The caves and karst of Texas. National Speleological Society, Huntsville, Alabama; 1994 p. 31–50.

[35] Trontelj P, Blejec A, Fišer C. Ecomorphological convergence of cave communities. Evolution 2012; 66(12):3852–65. 10.1111/j.1558-5646.2012.01734.x 23206142

[36] Beron P. Comparative study of the invertebrate cave faunas of Southeast Asia and New Guinea. Historia Naturalis Bulgarica 2015; 21: 169–210.

[37] Reboleira ASPS, Goncalves F, Oromí P, Taiti S. The cavernicolous Oniscidea (Crustacea: Isopoda) of Portugal. Eur J Taxon 2015; 1–61.

[38] Darda DM, Make DB. Osteological variation among extreme morphological forms in the Mexican Salamander genus *Chiropterotriton* (Amphibia: Plethodontidae): Morphological evolution and homoplasy. PLoS One 2015; 10 (6): e0127248 10.1371/journal.pone.0127248 26060996 PMC4464517

[39] Jeannel R. Les Fossiles vivants des caverns. Gallimard; 1949.

[40] Vandel A. Biospeleolgy. The biology of cavernicolous animals. Oxford, England, Pergamon; 1965.

[41] Rouch R, Danielopol DL. L’origine de la faune aquatique souterraine, entre le paradigme du refuge et le modèle de la colonisation active. Stygologia 1987; 3: 345–372.

[42] Deharveng L, Bedos A. Diversity patterns in the tropics In: White WB, Culver DC, editors. Encyclopedia of caves, 2nd ed. Academic/Elsevier; 2012 p. 238–250.

[43] Tian MY, Huang SB, Wang XH, Tang MR. Contributions to the knowledge of subterranean trechine beetles in southern China’s karsts: five new genera (Insecta, Coleoptera, Carabidae, Trechinae). ZooKeys 2016; (564): 121–156. PMCID: PMC4820093 10.3897/zookeys.564.6819 27081334 PMC4820093

[44] Zapparoli M. *Lithobius nuragicus* n. sp., a new *Lithobius* from a Sardinian cave (Chilopoda, Lithobiomorpha). Int J Speleol 1997; 25 (1): 59–66.

[45] Ázara LN, Ferreira RL. The first troglobitic *Cryptops* (*Trigonocryptops*) (Chilopoda: Scolopendromorpha) from South America and the description of a non-troglobitic species from Brazil. Zootaxa 2013; 3709 (5): 432–444. 26240920 10.11646/zootaxa.3709.5.2

[46] Stoev P, Akkari N, Komerički A, Edgecombe GD, Bonato L. At the end of the rope: *Geophilus hadesi* sp. n.–the world’s deepest cave-dwelling centipede (Chilopoda, Geophilomorpha, Geophilidae). ZooKeys 2015; (510): 95–114. 10.3897/zookeys.510.9614 26257537 PMC4523767

[47] Shear WA. Millipedes (Diplopoda) from caves in Mexico, Belize and Guatemala III. Problemi Attuali di Scienza e di Cultura, Quaderno Accademia Nazionale dei Lincei 1977; 171 (3): 235–265.

[48] Mauriès JP. Myriapoda (centipedes and millipedes) In: Gunn J, editor. Encyclopedia of caves and Karst Science. Taylor & Francis; 2004 p. 534–536.

[49] Reboleira ASPS, Enghoff H. Millipedes (Diplopoda) from caves of Portugal. J Caves Karst Stud 2014; 76 (1): 20–25.

[50] Golovatch SI. Cave Diplopoda of southern China with reference to millipede diversity in Southeast Asia. ZooKeys 2015; 510: 79–94. 10.3897/zookeys.510.8640 PMC4523766 26257536

[51] Loria SF, Zigler KS, Lewis JJ. Molecular phylogeography of the troglobiotic millipede *Tetracion* Hoffman, 1956 (Diplopoda, Callipodida, Abacionidae). Int J Myriap 2011; 5: 35–48.

[52] Culver DC, Shear WA. Myriapods. Encyclopedia of caves. Academic Press, Chennai 2012; 538–542.

[53] David JF. "Diplopoda–ecology". In: Minelli A. editor. Treatise on Zoology–Anatomy, Taxonomy, Biology. The Myriapoda 2. 2015; (12): 303–327.

[54] Howes C. Index to the biological supplements and records of the cave research group of Great Britain. Proc Univ Bristol Spelaeol Soc 1994; 20 (1): 15–41.

[55] Tabacura I, Giurginca A, Vànoaica L. Cavernicolous diplopoda of Romania. Travaux de l’Institut de Spéologie “Emile Racovitza 2003; 121–148.

[56] Beron P, Petrov B, Stoev P. The invertebrate cave fauna of the Western Rhodopes (Bulgaria and Greece) In: Beron P. editor. Biodiversity of Bulgaria 4. Biodiversity of Western Rhodopes (Bulgaria and Greece) II. Pensoft & Nat Mus Natur Hist Sofia; 2011 p. 583–662.

[57] Enghoff H, Reboleira ASPS. A new cave-dwelling millipede of the genus *Scutogona* from central Portugal (Diplopoda, Chordeumatida, Chamaesomatidae). Zootaxa 2013b; 3736 (2): 175–186. 25112621 10.11646/zootaxa.3736.2.5

[58] Hoffman RL. New genera and species of cavernicolous diplopods from Alabama. Alabama Geological Survey Museum Paper 1956; 35: 5–11.

[59] Causey NB. Millipedes in the collection of the Association for Mexican Cave Studies (Diplopoda). AMCS Bull. 1971; 4: 23–32.

[60] Iniesta LM, Ferreira RL, Wesener T. The first troglobitic *Glomeridesmus* from Brazil, and a template for a modern taxonomic description of Glomeridesmida (Diplopoda). Zootaxa 2012; 3550: 26–42.

[61] Deharveng L. Diversity in the tropics In: Culver DC, White A. editors, Encyclopedia of Caves. Academic Press, San Diego; 2005 p. 166–170.

[62] Hoffman RL. Studies on spiroboloid millipedes. XVIII. *Speleostrophus nesiotes*, the first known troglobitic spiroboloid millipede, from Borrow Island, Western Australia. Myriapodologica 1994; 3 (3): 19–24.

[63] Mauriès JP. *Guizhousma latellai* gen. n, sp. n., de Chine continentale, tyoe d’une nouvelle famille de la superfamille des Neoatractosomatoidea (Diplopoda: Chordeumatida). Arthropoda Sel 2005; 14: 11–17.

[64] Stoev P, Enghoff H. A new cave-dwelling millipede of the genus *Bollmania* Silvestri, 1896 from Yunnan, China, with remarks on the reduction of the second female leg-pair (Diplopoda: Callipodida: Caspiopetalidae). J Nat Hist 2005; 39 (21): 1875–1891.

[65] Lewis JJ. Six new species of *Pseudotremia* from caves of the Tennessee Cumberland Plateau (Diplopoda: Chordeumatida: Cleidogonidae). Zootaxa 2005; 1080: 17–31.

[66] Golovatch SI, Geoffroy JJ, Mauriès JP, VandenSpiegel D. Review of the millipede genus *Glyphiulus* Gervais, 1847, with descriptions of new species from Southeast Asia (Diplopoda, Spirostreptida, Cambalopsidae). Part 1: the *granulatus*-group. Zoosystema 2007a; 29 (1): 7–49.

[67] Golovatch SI, Geoffroy JJ, Mauriès JP, VandenSpiegel D. Review of the millipede genus *Glyphiulus* Gervais, 1847, with descriptions of new species from Southeast Asia (Diplopoda, Spirostreptida, Cambalopsidae). Part 2: the *javanicus*-group. Zoosystema 2007b; 29 (3): 417–456.

[68] Shear WA, Taylor SJ, Wynne JJ, Krejca JK. Cave millipeds of the United States. VIII. New genera and species of polydesmidan millipeds from caves in the southwestern United States (Diplopoda, Polydesmida, Macrosternodesmidae). Zootaxa 2009; 2151: 47–65.

[69] Tabacaru I. Dezovoltarea postembrionara la specii cavernicole de Gervaisia (Diplopoda, Gervaisiidae). Lucrarile Institutului de Speologie “E. Racovitza 1963; 1 (2): 341–99.

[70] Enghoff H. Modified mouthparts in *Hydrophilous* cave millipedes (Diplopoda). Bijdragen tot de Dierkunde 1985a; 55 (1): 66–67.

[71] Enghoff H. A new species of *Trogloiulus* with modified mouthparts. With a revised key to the species and new records of the genus (Diplopoda, Julida, Julidae). Lavori. Soc. Ven. Sc. Nat 1985b; 10: 69–77.

[72] Golovatch SI, Enghoff H. Pill-millipedes of the Canary Islands: the *Glomeris alluaudi*-group (Diplopoda, Glomeridae). Vieraea 2003; 31: 9–25.

[73] Stoev P. The first troglomorphic species of the millipede genus *Paracortina* Wang & Zhang, 1993 from south Yunnan, China (Diplopoda: Callipodida: Paracortinidae). Zootaxa 2004; 441: 1–8.

[74] Geoffory JJ, Golovatch SI. Some polydesmidan millipedes form caves in southern China (Diplopoda: Polydesmida), with description of four new species. Arthropoda Sel 2004; 13 (1–2): 19–28.

[75] Mauriès JP. *Guizhousma latellai* gen. n, sp. n., de Chine continentale, tyoe d’une nouvelle famille de la superfamille des Neoatractosomatoidea (Diplopoda: Chordeumatida). Arthropoda Sel 2005; 14: 11–17.

[76] Golovatch SI, Geoffroy JJ. Review of the Southeast Asian millipede genus *Pacidesmus* Golovatch, with the descripition of a new troglobitic species form southern China (Diplopda: Polydesmida: Polydesmidae). Zootaxa 2006; 1325: 363–368.

[77] Golovatch SI, Geoffroy JJ, Mauriès JP. Several new or poorly-known cavernicolous millipedes (Diplopoda) from southern China. Arthropoda Sel. 2006a; 15 (2): 81–89.

[78] Golovatch SI, Geoffroy JJ, Mauriès JP. Four new Chordeumatida (Diplopoda) from caves in China. Zoosystema 2006b; 28 (1): 75–92.

[79] Golovatch SI, Geoffroy JJ, Mauriès JP, VandenSpiegel D. Review of the millipede genus *Eutrichodesmus* Silvestri, 1910 (Diplopoda, Polydesmida, Haplodesmidae), with description of new species. ZooKeys 2009a; 12: 1–46. 10.3897/zookeys.505.9862 PMC4453233 26052236

[80] Golovatch SI, Geoffroy JJ, Mauriès JP. Two new species of the millipede genus *Desmoxytes* Chamberlin, 1923 (Diplopoda: Polydesmida: Paradoxosomatidae) from caves in southern China. Arthropoda Sel 2010; 19 (2): 57–61.

[81] Golovatch SI, Geoffroy JJ, Mauriès JP, VandenSpiegel D. Two new species of the millipede genus *Hypocambala* Silvesti, 1895 from China and Vietnam (Diplopoda: Spirostreptida: Cambalopsidae). Arthropoda Sel 2011a; 20 (3): 167–174.

[82] Golovatch SI, Geoffroy JJ, Mauriès JP, VandenSpiegel D. New species of the millipede genus *Glyphiulus* Gervais, 1847 from the *granulatus*-group (Diplopoda: Spirostreptida: Cambalopsidae). Arthropoda Sel 2011b; 20 (2): 65–114.

[83] Golovatch SI, Geoffroy JJ, Mauriès JP, VandenSpiegel D. New species of the millipede genus *Glyphiulus* Gervais, 1847 from the *javanicus*-group (Diplopoda: Spirostreptida: Cambalopsidae). Arthropoda Sel 2011c; 20 (3): 149–165.

[84] Golovatch SI, Liu WX, Geoffory JJ. Review of the millipede genus *Hyleoglomeris* Verhoeff, 1910 in China, with descriptions of new species (Diplopoda, Glomerida, Glomeridae). Zootaxa 2012; 3358: 1–27.

[85] Golovatch SI, Li YB, Liu WX, Geoffory JJ. Three new caverniccolous species of dragon millipedes, genus *Desmoxytes* Chamberlin, 1923, from southern China, with notes on a formal congener from the Philippines (Diplopoda, Polydesmida, Paradoxosomatidae). ZooKeys 2012; 185: 1–17. 10.3897/zookeys.185.3082 PMC3345791 22577310

[86] Golovatch SI, Geoffroy J-J, Mauriès J-P, VandenSpiegel D. Review of the millipede genus *Eutrichodesmus* Silvestri, 1910, in China, with descriptions of new cavernicolous species (Diplopoda, Polydesmida, Haplodesmidae). ZooKeys 2015; 505: 1–34. 10.3897/zookeys.505.9862 PMC4453233 26052236

[87] Liu WX, Tian MY. Four new cavernicolous species of the millipede genus *Eutrichodesmus* Silvestri, 1910 from southern China (Diplopoda: Polydesmida: Haplodesmidae). Zootaxa 2013; 3734 (2): 281–291. 25277912 10.11646/zootaxa.3734.2.11

[88] Liu WX, Golovatch SI, Tian MY. A review of the dragon millipede genus *Desmoxytes* Chamberlin, 1923 in China, with descriptions of four new species (Diplopoda: Polydesmida: Paradoxosomatidae). ZooKeys 2014; 448: 9–26. 10.3897/zookeys.448.8081 PMC4233391 25408607

[89] Liu WX, Tian MY. Two new cave-dwelling species of the millipede genus *Paracortina* Wang & Zhang, 1993 from southern China (Diplopoda, Callipodida, Paracortinidae). ZooKeys 2015a; 517: 123–140. 10.3897/zookeys.517.9949 PMC4547130 26312031

[90] Liu WX, Tian MY. A checklist of millipede genus *Hyleoglomeris* Verhoeff, 1910 in mainland China, with descriptions of seven new species (Diplopoda, Glomerida, Glomeridae). Zootaxa 2015b; 4032 (1): 103–116. 26624341 10.11646/zootaxa.4032.1.5

[91] Liu WX, Golovatch SI, Tian MY. Six new species of dragon millipedes, genus *Desmoxytes* Chamberlin, 1923, mostly from caves in China (Diplopoda, Polydesmida, Paradoxosomatidae). ZooKeys 2016; 557: 1–24. 10.3897/zookeys.577.7825 PMC4829881 27110186

[92] Shear WA. Five new chordeumatidan millipeds from China: new species of *Vieteuma* (Kashmireumatidae) and *Nepalella* (Megalotylidae). Proc Calif Acad Sci 2002; 53 (6): 63–72.

[93] Stoev P, Geoffroy JJ. Review of the millipede family Paracortinidae Wang & Zhang 1993 (Diplopoda: Callipodida). Acta Arachnologica 2004; 53 (2): 93–103.

[94] Golovatch SI, Geoffroy JJ, Mauriès JP, VandenSpiegel D. Review of the millipede family Haplodesmidae Cook, 1895, with descriptions of some new or poorly-known species (Diplopoda, Polydesmida). ZooKeys 2009b; 12: 1–53. 10.3897/zookeys.505.9862 PMC4453233 26052236

[95] Golovatch SI, Geoffroy JJ, Mauriès JP, VandenSpiegel D. Two new species of the millipede genus *Trichopeltis* Pocock, 1894 (Diplopoda: Polydesmida: Cryptodesmidae) from Vietnam and China. Arthropoda Sel 2010; 19 (2): 63–72.

[96] Golovatch SI, Geoffroy JJ, Mauriès JP, VandenSpiegel D. An unusual new species of the millipede genus *Glyphiulus* Gervais, 1847 from Borneo (Diplopoda: Spirostreptida: Cambalopsidae). Russ Entomol J 2012c; 21 (2): 133–137.

[97] Golovatch SI. Review of the millipede genus *Epanerchodus* Attems, 1901 in continental China, with descriptions of new species (Diplopoda, Polydesmidae). Zootaxa. 2014a; 3760 (2): 275–288. 24870082 10.11646/zootaxa.3760.2.7

[98] Golovatch SI. Two new and one little-known species of the millipede genus *Epanerchodus* Attems, 1901 from southern China (Diplopoda, Polydesmida, Polydesmidae). Fragmenta Faunistica 2014b; 56 (2): 157–166.

[99] Golovatch SI, Geoffroy JJ. On some new or poorly-known species of the millipede family Polydesmidae from southern China (Diplopoda: Polydesmida). Russ Entomol J 2014; 23 (2): 91–105.

[100] Blanke A, Wesener T. Revival of forgotten characters and modern imaging techniques help to produce a robust phylogeny of the Diplopoda (Arthropoda, Myriapoda). Arthropod Struct Dev 2014; 43 (1): 63–75. 10.1016/j.asd.2013.10.003 24184600

[101] Enghoff H, Golovatch SI, Short M, Stoev O, Wesener T. "Diplopoda–taxonomic overview". In: Minelli A. editor. Treatise on Zoology–Anatomy, Taxonomy, Biology. The Myriapoda 2. 2015; (16): 363–453.

[102] Wesener T, VandenSpiegel D. A first phylogenetic analysis of Giant Pill-Millipedes (Diplopoda: Sphaerotheriida), a new model Gondwanan taxon, with special emphasis on island gigantism. Cladistics 2009; 25: 545–573. 10.1111/j.1096-0031.2009.00267.x 34879594

[103] Müller HG, Sombke A. "Diplopoda–sense organs". In: Minelli A. editor. Treatise on Zoology-Anatomy, Taxonomy, Biology. The Myriapoda 2. 2015; (9): 181–235.

[104] Shear WA. The chemical defenses of millipedes (Diplopoda): biochemistry, physiology and ecology. Biochem Syst Ecol 2015; 61: 78–117.

[105] Fudge DS, Szewciw LJ, Schwalb AN. Morphology and development of Blue Whale Baleen: An annotated translation of Tycho Tullberg's classic 1883 paper. Aquat Mamm 2009; 35 (2): 226–252.

[106] Buzilă R, Moldovan O. Antennal receptors in two representatives of Leptodirinae (Coleoptera, Cholevidae): diversity and adaptations. Evolution and Adaptation 2000; 6: 117–125.

[107] Culver DC, Holsinger JR, Christman MC, Pipan T. Morphological differences among eyeless amphipods in the genus *Stygobromus* dwelling in different subterranean habitats. J Crustacean Biol 2010; 30 (1): 68–74.

[108] Hopkin SP, Read HJ. The biology of millipedes. Oxford University, Oxford 1992.

[109] Wesener T, Enghoff H, Sierwald P. Review of the Spirobolida on Madagascar, with descriptions of twelve new genera, including three" fire millipedes"(Diplopoda). ZooKeys 2009; 19: 1–128.

[110] Hüppop K. Adaptation to low food In: White WB, Culver DC, editors. Encyclopedia of caves, 2nd ed. Academic/Elsevier; 2012; p. 1–9.

[111] Manton SM. The evolution of arthropodan locomotory mechanisms, part 4. The structure, habits and evolution of the diplopoda 1954; 299–368.

[112] Culver DC, Pipan T. Shiting paradigms of the evolution of cave life. Acta Carsologica 2015; 44(3):415–25.

[113] Taylor SJ. Cave adapted insects In: Capinera JL, editor. Encyclopedia of entomology. Springer Science and Business Media; 2008 p. 803–6.

[114] Köhler HR. Alberti G. Morphology of the mandibles in the millipedes (Diplopoda, Arthropoda). Zool Scr 1990; 19 (2): 195–202.

[115] Ishii K, Tamura H. A taxonomic study of polydesmoid millipedes (Diplopoda) based on their mandibular structures. Mémoires du Muséum National d’Histoire Naturelle 1996; 169: 101–111.

[116] Ashe JS. Mouthpart structure of *Stylogymnusa subantarctica* Hammond, 1975 (Coleoptera: Staphylinidae: Aleocharinae) with a reanalysis if the phylogenetic position of the genus. Zool J Linn Soc 2000; 130 (4): 471–498.

[117] Derkarabetian S, Steinmann DB, Hedin M. Repeated and Time-Correlated Morphological Convergence in Cave-Dwelling Harvestmen (Opiliones, Laniatores) from Montane Western North America. PLoS One 2010; 5 (5): e10388 10.1371/journal.pone.0010388 20479884 PMC2866537

[118] Hedin M, Thomas SM. Molecular systematics of eastern North American Phalangodidae (Arachnida: Opiliones: Laniatores), demonstrating convergent morphological evolution in caves. Mol Phylogenet Evol 2010; 54 (1): 107–121. 10.1016/j.ympev.2009.08.020 19699807

[119] Moldovan OT. Beetles In: White WB, Culver DC, editors. Encyclopedia of caves, 2nd ed. Academic/Elsevier; 2012 p. 54–62.

[120] Akkari N, Enghoff H, Metscher BD. A new dimension in documenting new species: high-detail imaging for myriapod taxonomy and first 3D cybertype of a new millipede species (Diplopoda, Julida, Julidae). PLoS One 2015; 10 (8): e0135243 10.1371/journal.pone.0135243 26309113 PMC4550252

[121] Juan C, Guzik MT, Jaume D, Cooper SJ. Evolution in caves: Darwin’s ‘wrecks of ancient life’ in the molecular era. Mol Ecol 2010; 19 (18): 3865–3880. 10.1111/j.1365-294X.2010.04759.x 20637049

[122] Stahl BA, Gross JB, Speiser DI, Oakley TH, Patel NH, Gould DB, et al A Transcriptomic Analysis of Cave, Surface, and Hybrid Isopod Crustaceans of the Species *Asellus aquaticus*. PLoS One 2015; 10 (10): e0140484 10.1371/journal.pone.0140484 26462237 PMC4604090

