# Supplementary material for: Convergent Evolution of Unique Morphological Adaptations to a Subterranean Environment in Cave Millipedes (Diplopoda)
Source: PLoS One. 2017 Feb 8;12(2):e0170717. doi: 10.1371/journal.pone.0170717 (PMC5298257; doi:10.1371/journal.pone.0170717)
Supplement: S1 Table — (DOCX) [file pone.0170717.s001.docx]

**S1 Table. Male characters.** **Note.** l/w: length/width ratio; ?: unknown; -----: absent. Abbreviations: ***Tro*** = troglobitic speices; ***Epi*** = epigean species; Pl = pectinate lamellae; iA = intermediate area; Mp = molar plate.

| **No.** | **Speices/Characters** | **Glomerida** | | **Polydesmida 1** | | **Polydesmida 2** | | **Polydesmida 3** | | **Chordeumatida** | | **Spirostreptida** | |
| --- | --- | --- | --- | --- | --- | --- | --- | --- | --- | --- | --- | --- | --- |
|  |  | ***Hyleoglomeris*** | | **Paradoxosomatidae** | | **Polydesmidae** | | **Haplodesmidae** | | ***Nepalella*** | | ***Glyphiulus*** | |
|  |  | ***Tro*** | ***Epi*** | ***Tro*** | ***Epi*** | ***Tro*** | ***Epi*** | ***Tro*** | ***Epi*** | ***Tro*** | ***Epi*** | ***Tro*** | ***Epi*** |
| 1 | Body with color/pigmentation | pallid | brownish | pink-  brownish | bright red | pallid | blackish | pallid | light-  brownish | pallid | brownish | light grey-brownish | yellowish, dark-brownish |
| 2 | Body length (mm) | 9.5 mm | ? | 30.3 mm | 19.2 mm | 21.5 mm | 20.4 mm | 12.1 mm | 5.9 mm | 23.9 mm | 22.7 mm | 32.9 mm | 28.2 mm |
| 3 | Midbody width (mm) | 5.4 mm | 4.7 mm | 2.7 mm | 1.9 mm | 2.6 mm | 3.5 mm | 2.7 mm | 1.1 mm | 2.0 mm | 2.4 mm | 1.4 mm | 1.7 mm |
| 4 | No. of ocelli | 6+1 | 8+1 | ----- | ----- | ----- | ----- | ----- | ----- | 5 / 5 | 22 / 25 | none? | 12 / 13 |
| 5 | Color of ocelli | brownish | blackish | ----- | ----- | ----- | ----- | ----- | ----- | transparent | brownish | ? | brownish to blackish |
| 6 | Size of ocelli | small | 2.0 times larger | ----- | ----- | ----- | ----- | ----- | ----- | small | small | hardly visible | small |
| 7 | Tömösváry organ (l/w) | 1.88 | 1.39 | ----- | ----- | ----- | ----- | ----- | ----- | ----- | ----- | ----- | ----- |
| 8 | Antennomere 3 (l/w) | 2.85 | 2.26 | 15.40 | 4.71 | 4.27 | 3.56 | 2.50 | 1.45 | 9.13 | 10.21 | 1.69 | 1.52 |
| 9 | Antennomere 4 (l/w) | 1.44 | 1.16 | 18.24 | 5.06 | 2.37 | 1.96 | 2.07 | 1.07 | 12.04 | 6.46 | 1.66 | 1.70 |
| 10 | Antennomere 5 (l/w) | 1.26 | 1.06 | 17.79 | 4.71 | 3.02 | 3.96 | 1.92 | 0.88 | 14.33 | 9.54 | 1.74 | 1.64 |
| 11 | Antennomere 6 (l/w) | 2.66 | 2.0 | 8.29 | 3.08 | 2.23 | 2.40 | 1.60 | 1.22 | 3.57 | 4.04 | 0.74 | 0.98 |
| 12 | Antennomeres 3–6 (l/w) | 2.09 | 1.63 | 14.83 | 4.34 | 2.87 | 2.89 | 1.96 | 1.14 | 9.14 | 7.32 | 1.43 | 1.66 |
| 13 | Antennomere 6 maximal width | near tip | at middle | near tip | near tip | near tip | near tip | near tip | near tip | near tip | near tip | near tip | near tip |
| 14 | Antenna apical cones | long | long | long | long | long | long | long | long | long | long | short | long |
| 15 | Labrum tooth | 1 | 1 | 3 | 3 | 3 | 3 | 3 | 3 | 3 | 3 | 3 | 5 |
| 16 | Mandible, external tooth | 1 | 1 | 1 | 1 | 1 | 1 | 1 | 1 | 1 | 1 | 1 | 2 |
| 17 | Mandible, no. of cusps of internal tooth | 4 | 4 | 4 | 4 | 4 | 4 | 4 | 4 | 6 | 6 | 5 | 7 |
| 18 | Mandible, Pl+iA/Mp | 1.27 | 1.05 | 1.57 | 1.10 | 0.78 | 1.45 | 1.43 | 1.19 | 1.73 | 1.08 | 1.62 | 1.55 |
| 19 | Collum (l/w) | 0.40 | 0.45 | 0.32 | 0.49 | 0.42 | 0.40 | 0.49 | 0.56 | 0.58 | 0.54 | 0.49 | 0.58 |
| 20 | Development of collum crests | ----- | ----- | ----- | ----- | ----- | ----- | ----- | ----- | ----- | ----- | flat, nearly obliteratus | high and evident |
| 21 | Shape of the paraterga | ----- | ----- | long-  spiniform | wing-like | ----- | ----- | ----- | ----- | ----- | ----- | ----- | ----- |
| 22 | 3+3 setae on metaterga | ----- | ----- | ----- | ----- | ----- | ----- | ----- | ----- | short | long | ----- | ----- |
| 23 | Development of metazonae crests | ----- | ----- | ----- | ----- | ----- | ----- | ----- | ----- | ----- | ----- | conspicuous but low | strongly crested |
| 24 | Midleg, femur (l/w) | 4.18 | 3.05 | 20.50 | 6.11 | 3.48 | 1.66 | 7.0 | 2.26 | 10.39 | 5.51 | 5.67 | 2.74 |
| 25 | Midleg, postfemur (l/w) | 0.78 | 0.55 | 3.78 | 2.80 | 0.89 | 0.89 | 0.68 | 0.44 | 2.62 | 1.98 | 1.77 | 1.28 |
| 26 | Midleg, tibia (l/w) | 0.88 | 1.0 | 24.30 | 3.84 | 1.29 | 1.0 | 1.16 | 1.03 | 2.56 | 1.92 | 1.94 | 1.25 |
| 27 | Midleg, tarsus (l/w) | 10.76 | 9.02 | 31.68 | 11.83 | 9.56 | 5.98 | 8.61 | 6.83 | 18.27 | 9.05 | 7.34 | 4.05 |
| 28 | Midleg, claw (l/w) | 8.04 | ? | 2.83 | 6.34 | 9.34 | 5.57 | 4.66 | 2.46 | 11.88 | 8.19 | 8.72 | 7.68 |
| 29 | Midleg, claw length/accessory spine length | ----- | ----- | ----- | ----- | ----- | ----- | ----- | ----- | ----- | ----- | 8.05 | 5.52 |
| 30 | Pre-anal crest | ----- | ----- | ----- | ----- | ----- | ----- | ----- | ----- | ----- | ----- | without crest | with an axial elevation |
