# Supplementary material for: Convergent Evolution of Unique Morphological Adaptations to a Subterranean Environment in Cave Millipedes (Diplopoda)
Source: PLoS One. 2017 Feb 8;12(2):e0170717. doi: 10.1371/journal.pone.0170717 (PMC5298257; doi:10.1371/journal.pone.0170717)
Supplement: S2 Table — (DOCX) [file pone.0170717.s002.docx]

**S2 Table. Female characters.** **Note.** l/w: length/width ratio; ?: unknown; -----: absent. Abbreviations: ***Tro*** = troglobitic speices; ***Epi*** = epigean species. Pl = pectinate lamellae; iA = intermediate area; Mp = molar plate.

| **No.** | **Species/Characters** | **Glomerida** | | **Polydesmida 1** | | **Polydesmida 2** | | **Polydesmida 3** | | **Chordeumatida** | | **Spirostreptida** | |
| --- | --- | --- | --- | --- | --- | --- | --- | --- | --- | --- | --- | --- | --- |
|  |  | ***Hyleoglomeris*** | | **Paradoxosomatidae** | | **Polydesmidae** | | **Haplodesmidae** | | ***Nepalella*** | | ***Glyphiulus*** | |
|  |  | ***Tro*** | ***Epi*** | ***Tro*** | ***Epi*** | ***Tro*** | ***Epi*** | ***Tro*** | ***Epi*** | ***Tro*** | ***Epi*** | ***Tro*** | ***Epi*** |
| 1 | Body with color/pigmentation | pallid | brownish | pink-  brownish | bright red | pallid | blackish | pallid | nearly pallid | pallid | brownish | light grey-brownish | yellowish, dark-brownish |
| 2 | Body length (mm) | 7. 6 mm | 8.6 mm | 27.4 mm | 21.0 mm | 26.6 mm | 23.1 mm | 10.4 mm | 5.9 mm | 25.3 mm | 23.9 mm | 40.6 mm | 30.1 mm |
| 3 | Midbody width (mm) | 4.3 mm | 4.8 mm | 2.4 mm | 2.7 mm | 3.3 mm | 4.5 mm | 2.2 mm | 0.9 mm | 2.3 mm | 2.9 mm | 1.7 mm | 1.9 mm |
| 4 | No. of ocelli | 5+1 | 7+1 | ----- | ----- | ----- | ----- | ----- | ----- | 4 / 5 | 25 / 25 | none? | 13 / 14 |
| 5 | Color of ocelli | brownish | blackish | ----- | ----- | ----- | ----- | ----- | ----- | tansparent | brownish | ? | brownish to blackish |
| 6 | Size of ocelli | small | 2.0 times larger | ----- | ----- | ----- | ----- | ----- | ----- | small | small | hardly visible | small |
| 7 | Tömösváry organ (l/w) | 1.33 | 1.22 | ----- | ----- | ----- | ----- | ----- | ----- | ----- | ----- | ----- | ----- |
| 8 | Antennomere 3 (l/w) | 2.78 | 2.23 | 13.75 | 5.44 | 4.67 | 4.02 | 2.05 | 1.48 | 9.17 | 5.85 | 1.95 | 1.56 |
| 9 | Antennomere 4 (l/w) | 1.22 | 1.03 | 14.97 | 4.28 | 3.20 | 3.48 | 1.07 | 0.99 | 12.83 | 6.57 | 1.67 | 1.49 |
| 10 | Antennomere 5 (l/w) | 1.22 | 1.12 | 14.61 | 4.59 | 3.71 | 4.18 | 0.88 | 0.96 | 9.54 | 7.68 | 1.97 | 1.52 |
| 11 | Antennomere 6 (l/w) | 2.66 | 2.30 | 7.27 | 2.73 | 2.65 | 2.24 | 1.25 | 1.05 | 3.44 | 3.80 | 1.11 | 0.89 |
| 12 | Antennomeres 3–6 (l/w) | 1.87 | 1.69 | 12.47 | 4.15 | 3.48 | 3.37 | 1.62 | 1.09 | 8.55 | 5.84 | 1.66 | 1.36 |
| 13 | Antennomere 6 maximal width | near tip | at middle | near tip | near tip | near tip | near tip | near tip | near tip | near tip | near tip | near tip | near tip |
| 14 | Antennae apical cones | long | long | long | long | long | long | long | long | long | long | short | long |
| 15 | Labrum tooth | 1 | 1 | 3 | 3 | 3 | 3 | 3 | 3 | 3 | 3 | 3 | 5 |
| 16 | Mandible, external tooth | 1 | 1 | 1 | 1 | 1 | 1 | 1 | 1 | 1 | 1 | 1 | 2 |
| 17 | Mandible, no. of cusps of internal tooth | 4 | 4 | 4 | 4 | 4 | 4 | 4 | 4 | 6 | 6 | 7 | 7 |
| 18 | Mandible, Pl+iA/Mp | 1.26 | 1.05 | 0.90 | 1.39 | 1.31 | 1.21 | 1.28 | 1.10 | 1.02 | 1.68 | 1.80 | 1.86 |
| 19 | Collum (l/w) | 0.43 | 0.53 | 0.39 | 0.47 | 0.41 | 0.35 | 0.55 | 0.53 | 0.56 | 0.87 | 0.61 | 0.58 |
| 20 | Development of collum crests | ----- | ----- | ----- | ----- | ----- | ----- | ----- | ----- | ----- | ----- | flat, nearly obliteratus | high and evident |
| 21 | Shape of the paraterga | ----- | ----- | long-  spiniform | wing-like | ----- | ----- | ----- | ----- | ----- | ----- | ----- | ----- |
| 22 | 3+3 setae on metaterga | ----- | ----- | ----- | ----- | ----- | ----- | ----- | ----- | short | long | ----- | ----- |
| 23 | Development of metazonae crests | ----- | ----- | ----- | ----- | ----- | ----- | ----- | ----- | ----- | ----- | conspicuous but low | strongly crested |
| 24 | Midleg, femur (l/w) | 4.82 | 2.79 | 11.04 | 6.96 | 4.98 | 4.15 | 6.53 | 3.13 | 10.42 | 5.18 | 6.23 | 2.55 |
| 25 | Midleg, postfemur (l/w) | 0.88 | 0.65 | 2.82 | 1.94 | 1.19 | 1.10 | 0.77 | 0.52 | 1.44 | 1.70 | 1.81 | 1.23 |
| 26 | Midleg, tibia (l/w) | 1.32 | 1.08 | 18.26 | 3.44 | 1.89 | 1.44 | 1.21 | 0.85 | 2.37 | 1.46 | 2.09 | 1.48 |
| 27 | Midleg, tarsus (l/w) | 9.02 | 6.98 | 28.65 | 9.57 | 8.79 | 9.27 | 6.83 | 5.64 | 16.19 | 11.10 | 7.18 | 4.23 |
| 28 | Midleg, claw (l/w) | 9.07 | 6.16 | 3.63 | 5.95 | 12.84 | 10.42 | 7.81 | 4.33 | 6.18 | 6.42 | 6.68 | 6.46 |
| 29 | Midleg, claw length/accessory spine length | ----- | ----- | ----- | ----- | ----- | ----- | ----- | ----- | ----- | ----- | 9.87 | 3.83 |
| 30 | Pre-anal crest | ----- | ----- | ----- | ----- | ----- | ----- | ----- | ----- | ----- | ----- | without crest | with an axial elevation |
